# WHERE HAVE ALL THE ADVOCATES GONE?

**DOI:** 10.1093/geroni/igae098.2092

**Published:** 2024-12-31

**Authors:** Robyn Stone

**Affiliations:** LeadingAge, Washington, District of Columbia, United States

## Abstract

We face a serious challenge as we enter this presidential election season: the lack of a unified advocacy community to support a coherent national political platform that needs to be addressed around the aging of the U.S. population over the next 15 years and into the future. This presentation briefly summarizes the history of the modern advocacy movement to improve the lives of older adults who were more likely than any other age group to be living in poverty in the 1960s and 1970s. The advocacy organizations were strong and united, helping to secure huge public investments including Medicare and Medicaid, affordable senior housing and the establishment of an aging network. Strong champions on Capitol Hill and in the executive branch supported these policies and investments. Ironically, as the older adult population is living longer, and the baby boomer population has grown larger than those under age 18, the advocacy voice has either disappeared or has become diffuse and less powerful. We need new advocacy efforts that bring younger and older generations together to advocate for an aging policy agenda that both presidential candidates are forced to acknowledge and address. The climate change advocacy approach that calls for short and long-term solutions to benefit current and future generations may provide a good framework for an intergenerational aging movement. When generations across age, racial/ethnic identity and other diverse groups (including class) see they are all in this together, a collective fortitude can emerge.

